# Antiviral activity of extracellular vesicles derived from respiratory syncytial virus-infected airway epithelial cells

**DOI:** 10.3389/fimmu.2022.886701

**Published:** 2022-08-12

**Authors:** Tiziana Corsello, Yue Qu, Teodora Ivanciuc, Roberto P. Garofalo, Antonella Casola

**Affiliations:** ^1^ Department of Pediatrics, The University of Texas Medical Branch at Galveston (UTMB), Galveston, TX, United States; ^2^ Department of Microbiology and Immunology, The University of Texas Medical Branch at Galveston (UTMB), Galveston, TX, United States

**Keywords:** extracellular vesicles, viral infection, airways, RSV, epithelial cells

## Abstract

Respiratory syncytial virus (RSV) is a major cause of acute lower respiratory tract infections in children and elderly. No vaccine or effective treatment is currently available for RSV. Extracellular vesicles (EVs) are microvesicles known to carry biologically active molecules, including RNA, DNA and proteins (i.e. cargo). Viral infections can induce profound changes in EV cargo, and the cargo can modulate cellular responses of recipient cells. We have recently shown that EVs isolated from RSV-infected cells were able to activate innate immune response by inducing cytokine and chemokine release from human monocytes and airway epithelial cells, however, we did not investigate the potential antiviral contribution of EVs to a subsequent infection. The objective of this study was to assess the presence of innate immune mediators, including type I and III interferons (IFNs) in EVs released from airway epithelial cells infected with RSV, and their potential role in modulating viral replication in recipient cells. EV-derived from cells infected with RSV were associated with significant amounts of cytokine and chemokines, as well as IFN-β and -λ, compared to EVs isolated from mock-infected cells. Cells treated with RSV-EVs showed significantly lower levels of viral replication compared to untreated or mock-EV-treated RSV infected cells. Cellular pretreatment with Cerdulatinib, an IFN receptor signaling inhibitor, inhibited the antiviral activity of RSV-EVs in recipient airway epithelial cells. Furthermore, treatment of A549 cells with RSV-EVs induced the expression of IFN-dependent antiviral genes, supporting the idea that RSV-EVs exerts their antiviral activity through an interferon-dependent mechanism. Finally, we determined the concentrations of soluble and EV-associated IFN-β and IFN-λ in five nasopharyngeal secretions (NPS) of children with viral infections. There were significant levels of IFN-λ in NPS and NPS-derived EVs, while IFN-β was not detected in either of the two types of samples. EVs released from RSV-infected cells could represent a potential therapeutic approach for modulating RSV replication in the airways.

## Introduction

Respiratory syncytial virus (RSV) is a negative-sense single-stranded RNA virus belonging to the *Pneumoviridae* family (1). It is the single most important virus causing acute lower respiratory tract infections in children and a major cause of severe respiratory morbidity and mortality in the elderly (2). About 45% of hospital admissions and in-hospital deaths due to RSV occurred in children younger than 6 months (3) and 1.5 million RSV episodes in older adults in industrialized countries in 2015 (4). In addition to acute morbidity, RSV infection has been linked to both the development and the severity of asthma. No vaccine or effective treatment is currently available for RSV (5).

Extracellular vesicles (EVs) are a type of secretory vehicle released from cells and isolated from various bio-fluids as bronchial lavage, breast milk, blood, and saliva (6–10). EVs are characterized by diameter (size), and specific EV markers, including CD63, CD9, ALIX, and TSG101 (11). EVs contain nucleic acids, lipids, and proteins, known as the EV cargo, and have been shown to transfer their biologically active cargo between neighboring cells and to distant sites, therefore participating in cell-to-cell communication, inflammation, and disease pathogenesis (12, 13). Previous studies in selected viral infections have suggested that EVs can play an important role in viral pathogenesis, by contributing to viral replication and spread, as well as to modulation of virus-host cellular interactions (14–16). Conversely, other groups have shown a protective role of these vesicles, conferring host cell resistance to viral infections, as in the case of Dengue virus in an *in vitro* model of infection (17). Although it is known that infections can alter the molecular cargo associated with EVs (15), their role in viral replication and pathogenesis remains largely unexplored, particularly in the context of single stranded RNA viruses.

We have previously described that RSV infection of A549 cells is associated with changes in EV cargo, which are not a simple reflection of changes occurring within infected cells. Importantly, we showed that EVs isolated from RSV-infected cells can activate innate immune responses by inducing release of cytokine and chemokine from EV-exposed (i.e. recipient) monocytes and airway epithelial cells (18). In the present study, we investigated whether EVs released from airway epithelial cells infected with RSV would carry a specific cargo of cytokines, chemokines and IFNs and tested whether RSV-EVs affected viral replication of exposed/recipient cells that were subsequently infected. We found that EV-derived from cells infected with RSV were associated with significant levels of cytokine and chemokines, as well as IFN-β and -λ, compared to EVs isolated from uninfected cells. Moreover, recipient epithelial cells treated with RSV-EVs showed significantly lower levels of viral replication compared to untreated or mock-EV-treated RSV infected cells. Pretreatment of recipient cells with Cerdulatinib, an IFN receptor signaling inhibitor, inhibited the antiviral activity of RSV-EVs. Furthermore, treatment of A549 cells with RSV-EVs induced the expression of IFN-dependent antiviral genes, suggesting that RSV-EVs exert their antiviral activity *via* an interferon-dependent mechanism. Finally, we determined the concentrations of soluble and EV-associated IFN-β and IFN-λ in five nasopharyngeal secretions (NPS) of children with viral infections and found significant levels of IFN-λ, but not IFN-β in NPS and NPS-derived EVs.

## Materials and methods

### Cell cultures and RSV infection

Human airway epithelial cell line A549 (human alveolar type II cell line -American Type Culture Collection, USA) were cultured and maintained in F12K culture media, supplemented with 10% FBS (HyClone, GE Healthcare USA), 100 U/mL penicillin G, 100 μg/mL streptomycin and 2 mM glutamine. RSV stocks and viral pools were prepared as previously described (18, 19). Primary small airway epithelial (SAE) cells (Lonza Inc., San Diego, CA, USA), derived from the terminal bronchioli of cadaveric donors, were grown in culture medium containing 7.5 mg/mL bovine pituitary extract (BPE), 0.5 mg/mL hydrocortisone, 0.5 µg/mL hEGF, 0.5 mg/mL epinephrine, 10 mg/mL transferrin, 5 mg/mL insulin, 0.1 µg/mL retinoic acid, 0.5 µg/mL triiodothyronine, 50 mg/mL gentamicin, and 50 mg/mL bovine serum albumin. SAE cells were switched to basal media (no supplemented added) several hours prior to RSV infection. When A549 cells were used to isolate EVs, they were changed to exo-free FBS medium, 4 hours before and throughout the length of the experiment. At 90 to 95% confluence, cell monolayers were infected with RSV at multiplicity of infection (MOI) of 1. An equivalent amount of 30% sucrose solution was added to uninfected A549 or SAE cells as a control (mock cells). The culture media, from both mock and RSV-infected cells, were collected after 24 hours p.i. and processed for the next analyses. Viral titers were measured by plaque assay in HEp2 cells as described in (20).

### Extracellular vesicles isolation and purification

Culture media collected from 2 × 10^7^ mock-infected or RSV-infected cells (24 hours) and nasopharyngeal secretions (NPS) samples of patients were subjected to debris removal by centrifugation at 3,000 g for 15 min at 4°C. The clear media and NPS samples were subjected to further cleaning by filtration through 0.22 μm sterile filter to remove any remaining debris. The filtered media was transferred to Amicon^®^ Ultra-15 centrifugation filters (Millipore, Billerica, MA, USA) and centrifuged at 2500 g for 35 min. Exoquick-TC (System Biosciences, USA) reagent was added to the filtered media or NPS, mixed thoroughly, and incubated overnight at 4°C to precipitate EVs. Next morning the mixture was subjected to centrifugation at 1,500 g for 30 min, the EV pellets were washed and resuspended in filtered PBS. To remove contaminating viral particles, EVs were subjected to CD63 immuno-purification using CD63 exosome isolation reagents (System Biosciences, USA), following manufacturer’s instructions. The purified EVs were eluted from the bound CD63 beads in an average of 300 μl and used for experimental procedures. Protein concentration was determined using a protein assay kit from Bio-Rad, USA. Purified EVs from cells were screened for presence of replicating virus, to avoid using contaminated preparations. This screening was done by plaque assay, inoculating a fraction of the EV pool onto HEp2 cells.

### Extracellular vesicles characterization

EV size distribution and number of particles were analyzed using the NanoSight™ LM10-HS10 system (Malvern Instruments, UK). NanoSight™ tracking analysis (NTA) software was used to produce the mean and median vesicle size together with the vesicle concentration (in millions). Samples were measured 3 times to ensure reproducibility. The instrument was rinsed between samples using filtered water. EV markers were analyzed by Western Blot assays. EV samples were lysed in a buffer (50mM Tris NaCl, 0.5% Triton, 300 mM NaCl) supplemented with a protease and phosphatase inhibitor cocktail. Equal amount of protein, 15 µg in total, were processed as described previously (18). The primary antibodies for Western blot were rabbit anti-human CD63 (1:1000; System Biosciences), anti-human Alix (1:500; Santa Cruz), anti-human apoB (1:500; Novus Biologicals) and mouse anti-human GM130 cis –Golgi (1:800; Santa Cruz).

### Cytokine and chemokine analysis

Intact and lysed (pretreated with Triton 1%) EV samples were used to measure the levels of cytokine/chemokines and IFNs. EV fractions were quantified using the NanoSight instrument and normalized to the same particle number prior to IFN and cytokine assays. Cytokines and chemokines were measured using the Bio-Plex Cytokine Human Multi-Plex panel (Bio-Rad Laboratories, Hercules, CA) according to the manufacturer’s instructions. Immunoreactive IFN-β and IFN-λ 2/3 were measured using commercial enzyme-linked immunosorbent assays (ELISAs), following the manufacturer’s protocol (PBL Biomedical Laboratories, Piscataway, NJ, USA).

### EV treatments of cells

A549 cells were placed in 24-well plates and grown overnight. The next day the cell media was removed, cells were washed with PBS, and fresh media containing equal amounts of mock- or RSV- EVs (15 µg/well) was added. Mock- or RSV- EVs were isolated and purified using a two-step EV purification method, Exoquick-TC followed by CD63 immuno-EV purification. The cells were allowed to incubate in the presence of EVs for 24 hours. Negative control wells were included that consisted of mock or RSV-infected cells not treated with EVs. For inhibition of IFN signaling, cells were pretreated with 5 μM of Cerdulatinib (Selleckchem, TX) 1 h prior to and throughout the EV treatment. At the end of the EV treatment, media were removed, and cells were infected with RSV for 24h. After infection, media were collected from each well and stored at -80°C for further analyses.

### Reverse transcription - qPCR

RNA was extracted from A549 cells using an Aurum Total RNA Mini Kit (BioRad, Hercules, CA, USA) according to the manufacturer instructions. RNA samples were quantified using a DS-11 Spectrophotometer (DeNovix Inc., Wilmington, DE, USA). Synthesis of cDNA was performed with 1 μg of total RNA in a 20 μL reaction using iScript Reverse Transcription Supermix reagent according to the manufacturer’s instructions (BioRad, Hercules, CA, USA). PCR amplification was done using 1 μL of cDNA in a total volume of 25 μL using a SYBR Green Fast qPCR mix (ABclonal, Woburn, MA, USA). 18S RNA was used as a housekeeping gene for normalization. PCR assays were run in the BioRad CFX Connect Real-Time System. Triplicate CT values were analyzed in Microsoft Excel using the comparative CT (ΔΔCT) method. The amount of target (2−ΔΔCT) was obtained by normalizing the endogenous reference (18S) sample. Primer sequences of Mx1, DDX58 and ISG15 genes are available upon request.

### Western blot

Total cell lysates were prepared with RIPA buffer (Cell Signaling, 9806) and protein concentration was determined with Pierce BCA Protein Assay Kit (ThermoFisher, 23225). Equal amounts (15 μg) of proteins were separated by SDS-PAGE and transferred onto polyvinylidene difluoride (PVDF) membrane. Nonspecific binding was blocked by immersing the membrane in Tris-buffered saline-Tween (TBST) blocking solution containing 5% skim milk powder. After blocking, the membranes were incubated with the primary antibody overnight at 4°C, followed by the appropriate secondary antibody diluted in TBST for 1h at room temperature. Proteins were detected using enhanced chemiluminescence (ECL). The primary antibody used for phospho-STAT1 was from Cell Signaling, cat#9167S. β-Actin was used as loading control protein to normalize the target proteins expressions from whole cell extracts (Sigma-Aldrich, cat#A1978).

### Nasopharyngeal secretions samples collection

NPS were collected as part of an ongoing IRB-approved study on the pathogenesis of lower respiratory tract infections in children up to 2 years of age. After written informed consent was provided by the parent or legal guardian, NPS samples were collected from patients at the time of the visit in the ER, or within 24 hours after hospital admission. Control NPS were children admitted to the Pediatric Intensive Care Unit following surgery for conditions unrelated to airways disease and negative for viral infections. Samples were immediately transported to the lab in ice and tested for respiratory viruses, using the multiplex RT-PCR-based Luminex xTAG Respiratory Viral Panel (RVP, Luminex Molecular Diagnostics) to detect simultaneously 19 viral targets. An aliquot of NPS was used for direct analysis of IFN-β or IFN-λ 2/3 and another aliquot for the isolation of EVs as described in the previous section.

### Statistical analysis

A two-tailed Student’s t test using a 95% confidence level was performed in all experiments. Significance is indicated as a p value of <0.05 (*). Fold change of RT-PCR experiments was calculated by 2-ΔΔCt method and represent mean ± SEM using GraphPad Prism v4 (GraphPad Software).

## Results

### Cytokines and interferons in EVs derived from airway epithelial cells

**Figure 1:**
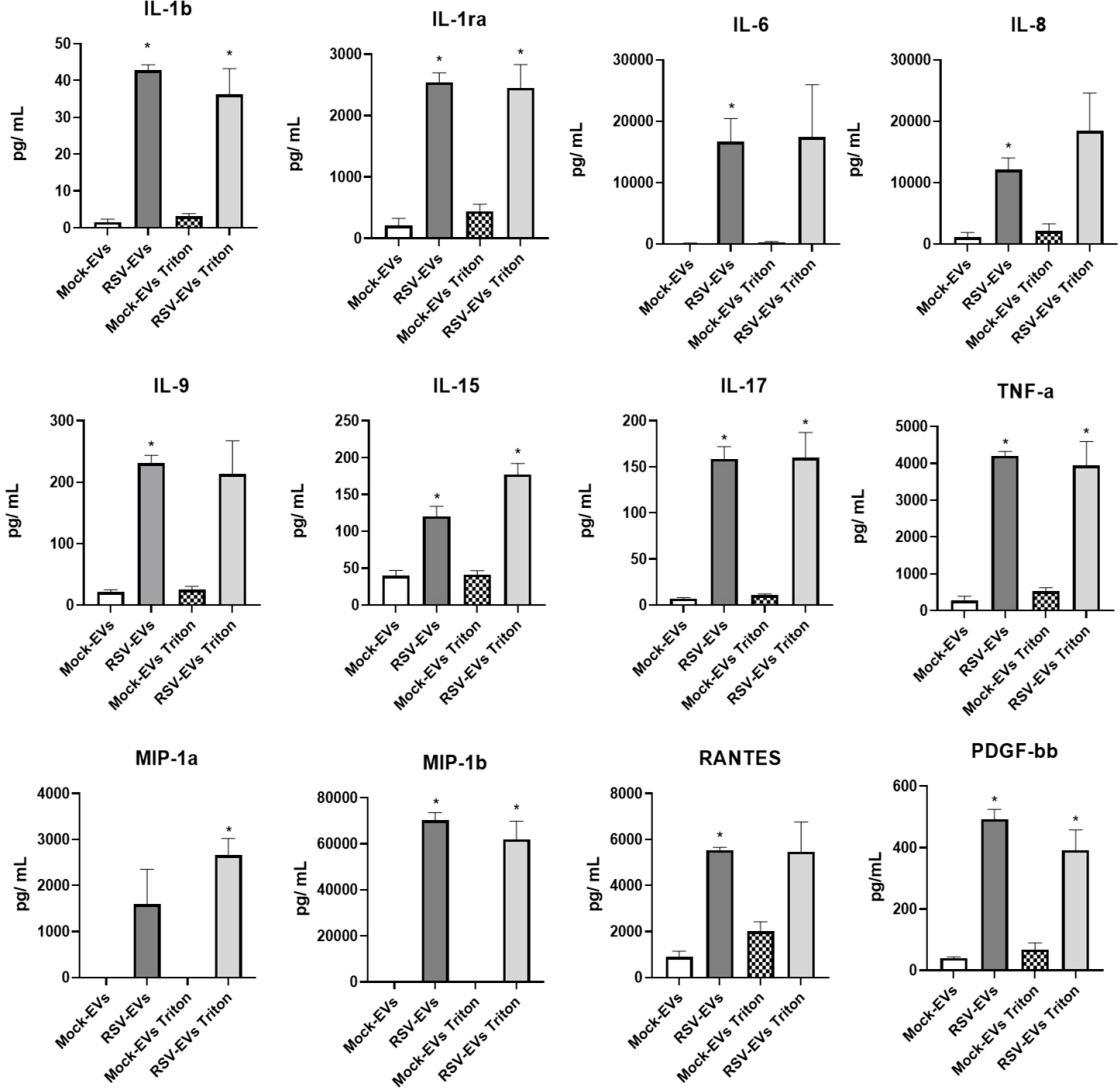
Cytokine concentrations associated with EVs released from mock- and RSV-infected A549 cells. Cytokines were measured using a human multi-plex panel array as intact or Triton lysed EVs. Data are presented as mean ± SEM. * indicates a statistical difference comparing RSV- EVs or RSV-Triton versus Mock-EVs or Mock-Triton, respectively (*p value < 0.05). Data represents the average of three independent experiments.

Studies over the last few years have identified EVs as a non-canonical mechanism by which cytokines can be secreted into the extracellular space and modulate functions of neighboring and distant cells. They can be membrane-associated or encapsulated withing the EVs, reviewed in (21), although the cytokine packaging mechanisms into EVs is not fully known. To determine whether immune mediators such as cytokines, chemokines and IFNs were associated with EVs released from airway epithelial cells infected with RSV, we first isolated and purified EVs from A549 cells using a two-step EV purification method, Exoquick-TC followed by CD63 immuno-EV purification as previously published from our laboratory (18). We used a precipitation reagent-based EV enrichment, followed by CD63 antibody based immuno-magnetic isolation, which results in EV preparations mostly devoid of RSV particles (18). Equal numbers of intact EVs derived from mock (mock-EVs) or RSV-infected cells (RSV-EVs), or EVs lysed with 1% Triton x solution (22), (mock-EVs Triton or RSV-EVs Triton), were used to measure surface-associated and encapsulated cytokines and chemokines by Bio-Plex Cytokine Multi-Plex array. Concentrations of IFN-β and IFN-λ in intact or lysed EVs were measured by ELISA. IL-1β, IL-1ra, IL-15, IL-17, TNF-α, MIP-1β and PDGF-bb were significantly increased in both RSV-EVs and RSV-EVs Triton purified from A549 cells, compared to mock-EVs or mock-EVs Triton. Significantly higher concentrations of IL-6, IL-8, IL-9 and RANTES were detected in RSV-EVs compared to mock-EVs, while MIP-1α concentration was significantly increased in RSV-EVs Triton compared to mock-EVs Triton (**Figure 1**).

**Figure 2:**
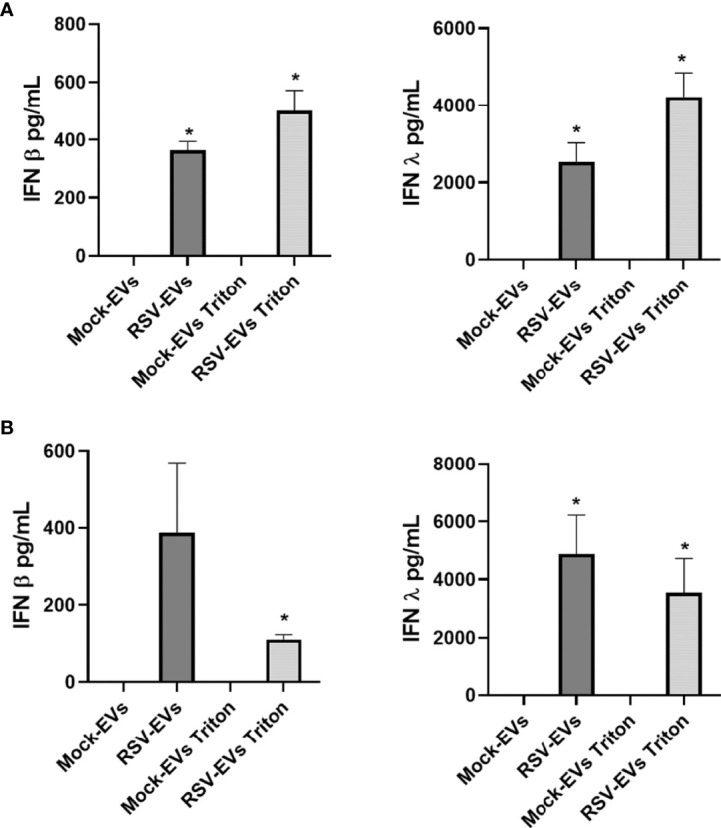
IFN-β and IFN-λ concentrations associated with EVs released from mock- and RSV-infected A549 **(A)** and SAE cells **(B)**. IFN-β and IFN-λ were measured by ELISA as intact or Triton lysed EVs. Data are presented as mean ± SEM. * indicates a statistical difference comparing RSV- EVs or RSV-EVs Triton versus Mock-EVs or Mock-EVs Triton, respectively (*p value < 0.05). Data represents the average of three independent experiments.

As type I and III IFNs secreted from infected cells represent the main host defense system against viral infections (23, 24), we also assessed IFN-β and IFN-λ levels in EVs isolated from A549 cells. We detected IFN-β and IFN-λ in intact and Triton lysed RSV-EVs, but not in mock-EVs (**Figure 2A**), with a trend of higher levels detected in Triton lysed EVs. We then confirmed the presence of IFNs in EVs released from normal human SAE cells, which showed results similar to A549 cells with the exception of levels of IFN-β associated with Triton lysed RSV-EVs, which was lower than the one present in intact EVs (**Figure 2B**).

### Antiviral activity of RSV-EVs on exposed cells occurs *via* an IFN-mediated mechanism

**Figure 3:**
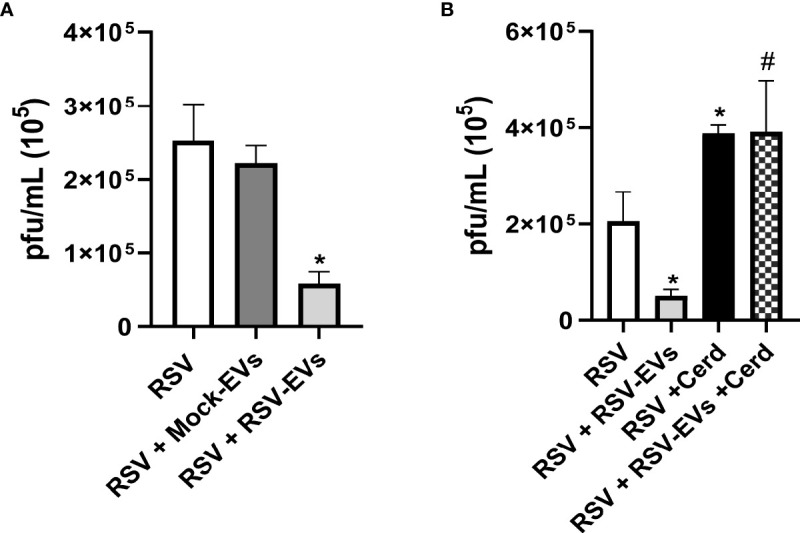
Effect of EV treatment on RSV replication. **(A)** A549 cells were infected with RSV alone (RSV) or treated with Mock-EVs (RSV+Mock-EVs) or RSV-EVs (RSV+RSV-EVs) (15 μg) for 24 hours and then infected with RSV (MOI of 1). **(B)** A549 cells were infected with RSV alone (RSV), pretreated with RSV-EVs (15 μg) 24 hours prior to RSV infection (RSV+RSV-EVs), infected with RSV and treated with Cerdulatinib (5 μM) 1h prior to infection (RSV-Cerd), or were pretreated with RSV-EVs for 24 hrs, treated with Cerd 1h prior to infection and then infected with RSV at a MOI of 1 (RSV+RSV-EVs+Cerd). Supernatants of infected cells were collected at 24 hours post-infection and viral titers were determined by plaque assay. Data are expressed as mean ± SEM. * indicates a statistical difference compared to RSV alone, while # indicates a difference between RSV-EV treated and RSV-EV treated plus Cerdulatinib groups (* or # for *p* ≤ 0.05). Data represents the mean average of three independent experiments.

To investigate whether EVs could exert an antiviral effect on recipient cells, A549 cells were pre-treated with 15 µg of mock or RSV-EVs for 24 hours, were then infected with RSV for 24 hours and harvested to collect cell supernatants to measure viral titers. In our previous study, we observed a functional effect in A549 cells treated with EVs (10 μg/well). This time, we increased and selected 15 μg/well EV-dose for a stronger anti-viral effect of EVs than the previous dose. We observed a significant decrease in RSV replication in cells treated with RSV-EV, compared to untreated and mock-EV treated infected cells (**Figure 3A**), indicating that RSV-EV cellular exposure confers protection against a subsequent infection. To determine whether the observed antiviral effect of RSV-EVs treatment was due to the presence of IFNs in RSV-EVs, recipient cells were treated with Cerdulatinib, an IFN receptor signaling inhibitor, prior to RSV-EV addition and subsequent infection. Cerdulatinib pretreatment resulted in loss of RSV-EV antiviral activity, with increased viral replication in recipient cells treated with the inhibitor, compared to recipient cells treated with RSV-EVs only, indicating that IFNs carried by RSV-EVs are biologically active (**Figure 3B**).

**Figure 4:**
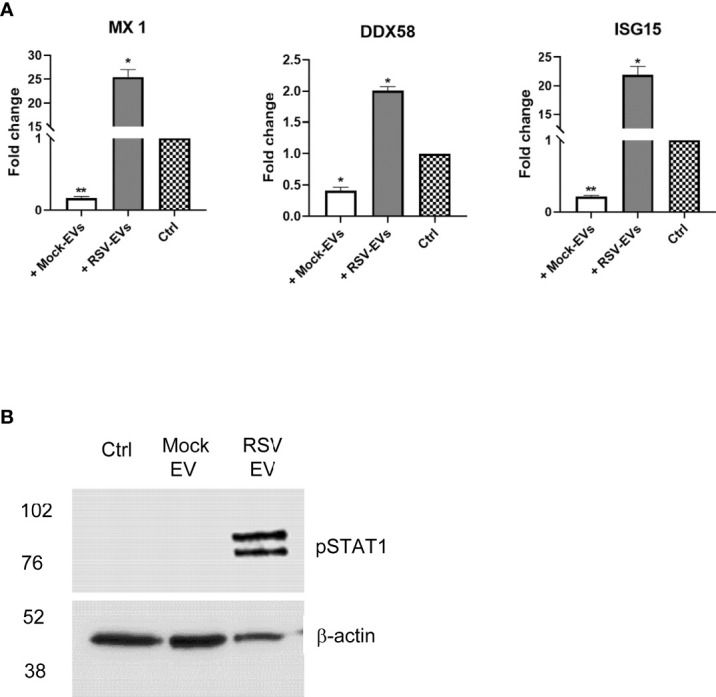
A549 cells were treated with Mock- or RSV- EVs (15 μg) for 24 hours and harvested to either extract total RNA or to prepare total cell lysates.A549 control cells with no EVs treatment are represented by checkered bar plot **(A)**. RNA extracted from A549 mock cells with or without EV treatment was subjected to RT-qPCR to measure the expression of antiviral genes MX1, DDX58 and ISG15. Fold changes in antiviral gene expressions were determined by 2-ΔΔCT method and represent mean ± SEM normalized to 18S. Cell treated with RSV- or Mock- EVs versus mock cells (*p value < 0.05; **p value < 0.01). Data represents the average of three independent experiments. **(B)**. Total cell lysates were subjected to western blot analysis using an antibody anti-phospho-STAT1 (pSTAT1). Membrane was stripped and re-probed with anti-β-actin for loading control. Western blot figure is representative of two independent experiments.

To confirm that RSV-EV exposure was associated with the induction of an antiviral gene response in recipient cells, A549 cells were treated with 15 µg of mock- or RSV-EVs for 24 hours and harvested to collect total RNA. Levels of the antiviral gene Mx1, DDX58 and ISG15 mRNA were assessed by RT-qPCR. We found that cell exposure to RSV-EVs induced a significant increase of Mx1, DDX58 and ISG15 gene expression, compared to basal levels in untreated (mock) cells (**Figure 4A**). Surprisingly, exposure to mock-EVs resulted in a significant inhibition of the basal levels of these antiviral genes. Engagement of both type I and III IFN receptors leads to activation of STAT1 and 2 proteins, through their tyrosine phosphorylation, which together with IRF9 form the ISGF3 complex necessary for induction of antiviral genes. To confirm activation of this pathway following RSV-EV treatment, A549 cells were treated with 15 µg of mock- or RSV-EVs for 24 hours and harvested to prepare total cell lysates. Levels of tyrosine phosphorylated STAT1 (pSTAT1) were then assessed by Western blot assay. Only stimulation of cells with RSV-EV led to activation of STAT1, with no phosphorylation induced by mock-EV (**Figure 4B**). Collectively, these results support the concept that RSV-EVs can exert an antiviral activity in exposed cells through an interferon-dependent mechanism.

### IFN content of EVs isolated from NPS of children with viral infections

**Figure 5:**
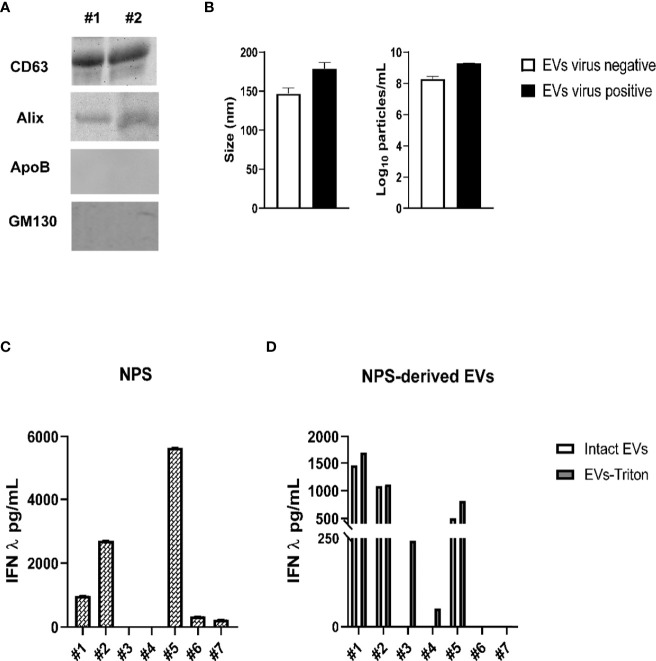
Characterization of NPS-derived EVs from children with and without viral infections. **(A)** Western blot analysis of equal amounts of purified EVs (5 μg) for CD63, Alix, ApoB and GM130. #1 and 2 indicates EV samples isolated from two representative patients. **(B)** Absolute size (left graph) and concentration (right graph) determined by Nanosight analysis of EVs isolated from virus negative (open bars) or virus positive (black bars) NPS. IFN-λ concentrations were measured by ELISA in NPS samples **(C)** or NPS-derived EVs **(D)** of children with or without viral lower respiratory tract infections. Patients #1 and #2 = RSV; Patient #3 = SARS-CoV-2; Patients #4 and #5 = Rhinovirus (RV), Patients #6 and #7 = Virus-negative.

We collected NPS from five children < 2 years of age who were admitted to the hospital for a lower respiratory tract infection or from two children negative for viral infections and who were admitted to the Pediatric Intensive Care Unit following surgery for conditions unrelated to airways disease (control sample). Presence of respiratory viruses was confirmed by the Luminex xTAG Respiratory Viral panel. Children were positive for RSV, rhinovirus (RV) and SARS-CoV-2. We isolated EVs from the NPS using the two-step purification protocol and we confirmed by immunoblot that they expressed the marker CD63 and Alix, and were negative for the Apolipoprotein B (ApoB) and cis-Golgi matrix protein GM130 (**Figure 5A**). EV size and particle number of NPS-derived EVs were measured by Nanosight instrument (**Figure 5B**). The average size of NPS-derived EVs of virus positive patients was 170 nm, and the average number of particles was 1.95 × 10^9^ particles/mL, while the average size of EV from control patients was 145 nm, with an average number of particles of 2.1 × 10^8^ particles/mL.

Next, we determined the concentrations of IFN-β and IFN-λ in NPS (**Figure 5C**) and NPS-derived EVs (**Figure 5D**) of these seven children. While IFN-β levels were below the limit of detection in both NPS and NPS-derived EVs of all children, IFN-λ was detected in five of the seven NPS samples (patients #3 and #4 had values below the limit of detection of the assay), with values ranging from 300 to 5,160 pg/mL. In addition, significant levels of IFN-λ were found to be associated with five NPS-derived EVs (upper values, 1693 pg/mL) from infected patients, excepted for the two patients with no viral infections (patients #6 and #7). Slightly higher concentrations of IFN-λ were detected in lysed (Triton-treated) EVs compared to intact EVs derived from those viral infected NPS samples with detectable IFN-λ, while we found IFN-λ only in the Triton lysed but not intact EVs isolated from the two infected NPS sample. EVs isolated from the airway secretions of two “control” infants negative for viral infections showed no detectable levels of IFN-λ in intact or Triton lysed EVs. Overall, our results suggest that IFN-λ is associated with EVs isolated from airway secretions during episodes of viral respiratory infections.

## Discussion

Each year in the United States, RSV leads to approximately 58,000 and 177,000 hospitalizations of children younger than 5 years old and adults aged 65 years or older, respectively (25). No effective drug or vaccine is currently available for RSV (5). There is a huge demand to develop therapeutic approaches and vaccines to treat respiratory viral infections such as RSV. The aims of this study were to 1) evaluate the innate immune mediator cargo of EVs derived from airway epithelial cells with RSV infection, and 2) investigate the potential antiviral activity of EVs isolated from infected airway epithelial cells.

In our previous published work, we isolated, purified, and characterized EVs from airway epithelial cells. We found significantly higher levels of cellular RNA species, named small non-coding RNAs, in EVs than in infected cells. EVs isolated from infected RSV cells carried viral RNAs as well as selected viral proteins, although they were not able to transmit infection to uninfected cells. These previous results underlined the changes of EV-cargo associated with RSV infection (18). In the current study, we show for the first time that EV-derived from cells infected with RSV carry a significant amount of cytokine, chemokines and IFN cargo, compared to EVs isolated from mock-infected cells. In the past few years, there has been a growing literature indicating that EVs can function as alternative carriers for the delivery of cytokines and chemokines, specifically these mediators can be packaged into microvesicles released from cells or can be secreted in membrane-bound form through vesicles-like exosomes, reviewed in (21). In a comprehensive study of cytokine association with EVs, Fitzgerald and colleagues have shown that that cytokine encapsulation into EVs can be found *in vitro*, *ex vivo* and *in vivo* biological experimental models, and that encapsulation in EVs is not associated with a specific cytokine but rather with the specific system and stimulus investigated (22). The same authors also provided evidence that the EV-associated cytokines were biologically active, by using reporter cell lines that needed specific cytokines to proliferate (22). EV-associated cytokines have been shown to possess a wide range of functions in multiple biological processes. For instance, in HIV-infected individuals, plasma-derived exosomes were highly enriched in a variety of cytokines, and exposure to these exosomes resulted in the induction of CD38 expression on naive and memory CD4+ and CD8+ T cells, a mechanism that could contribute to HIV propagation *via* bystander cell activation (26). Obregon *et al.* showed that EVs derived from lipopolysaccharide (LPS)-activated dendritic cells are important carriers of tumor necrosis factor (TNF)-α, which is important for the induction of proinflammatory mediators from epithelial cells upon internalization (27). In the cancer literature, transforming growth factor β (TGF-β) associated with tumor-derived EVs has been shown to promote tumor progression by stimulating the migration of cancer cells, by inhibiting T-cell responses, and by inducing differentiation of fibroblasts into myofibroblasts that support tumor growth, vascularization, and metastasis, reviewed in (21).

In this study, we found that both IFN-β and IFN-λ were present in significant concentrations in EVs released from RSV-infected cells, while no measurable levels of IFNs were found in EVs originated from uninfected A549 and SAE cells (**Figure 2**). Treatment with Triton resulted in some increase in the detected levels of IFNs in RSV-infected A549, but not in SAE cells (with even less IFN-β in Triton-treated compared to untreated ones). We do not know at this point the reason for this finding, although we can speculate that treatment of EVs with Triton, which is a milder detergent compared to other chemicals, may not lead to a full lysis of SAE-derived EVs, may interfere with the measurement of IFNs, or affect some of the experimental steps that precede the measurement of these mediators. Nonetheless, future studies will address the question regarding the relative distribution of cytokines and IFNs within the epithelial cell EVs, in particular those mediators that are surface-bound or encapsulated (22).Both type I and III IFNs are expressed in a variety of epithelial cells and released in response to viral infections, to induce an antiviral state in host cells (23, 28–30). IFN-λ was also present in EVs derived from NPS samples of children with viral respiratory infections, while there was no detectable IFN-β either in the NPS-EVs or in the originating samples. We recognize that our data are representative of a small group of young patients or controls purposely selected based on different viral pathogens that were associated with episodes of lower respiratory tract infections. Thus, this study was not designed to address statistical differences in EV-associated IFNs levels between viral pathogens or other clinical parameters, rather to extend our *in vitro* observation to human-derived samples of airway EVs. Although larger clinical studies that will include a diverse spectrum of viral respiratory pathogens will be necessary to confirm our findings, recent elegant studies using nasal cell organoids have shown that RSV is indeed a strong inducer of IFN-λ (31). The biological significance of our discovery was supported by: 1) evidence that exposure of recipient cells to RSV-EVs was associated with significantly reduced replication of a subsequent RSV infection; 2) pre-treatment with an IFN receptor signaling inhibitor abolished this protective effect; and 3) treatment of recipient cells with RSV-EVs induced activation of STAT1 protein and a significant increase of IFN-inducible antiviral genes Mx1, DDX58 and ISG15, which have been shown to control RSV replication (32). These data altogether support the idea that IFNs carried by epithelial RSV-EVs function as messengers to help blocking viral replication in neighbor cells.

Although initial studies have shown that EVs may promote pathogen transmission and spreading of viral infections (HCV/HIV) (33–35), other studies demonstrate on the contrary that EVs have a protective role by limiting viral replication, as it has been shown for Dengue virus (17), Rift Valley Fever virus (36) and influenza virus in a rodent model of infection (37). Our study reports for the first time a significant association of cytokines, chemokines and IFNs with EVs released from airway epithelial cells following RSV infection. Furthermore, our finding of type III IFN associated with EVs isolated from respiratory secretions of children infected with respiratory viruses support the concept that the packaging of innate immune mediators in EVs could be indeed an important mechanism to modulate innate and antiviral responses both close and far from the site of initial viral entry or infection. These mediators can be concentrated within EVs and exert their activity at the surface of other cells that might not otherwise be targeted by cytokines in soluble, circulating form (22). Also, the lipid bilayer structure of EVs has been shown to protect the antiviral molecules from extracellular degradation during the cell-to-cell communication *via* EVs and facilitate cytokine delivery and targeting to distant cells (38). Better understanding of the cargo and antiviral and immunomodulatory properties of EVs released from airway epithelial cells following respiratory virus infection could provide insight into the regulation of viral-induced responses and be the basis for potential EV-mediated antiviral strategies to treat/prevent infections.

## Data availability statement

The original contributions presented in the study are included in the article/supplementary material. Further inquiries can be directed to the corresponding authors.

## Ethics statement

The studies involving human participants were reviewed and approved by IRB 03-117 UTMB (The University of Texas Medical Branch). Written informed consent to participate in this study was provided by the participants’ legal guardian/next of kin. Written informed consent was obtained from the minor(s)’ legal guardian/next of kin for the publication of any potentially identifiable images or data included in this article.

## Author contributions

TC designed and performed the experiments, analyzed the data, wrote the paper; YQ and TI contributed data to the viral titers or interferons/cytokines studies; TC, AC and RG acquired the listed funding, conceived, designed the experiments, and finalized the manuscript. All authors contributed to the article and approved the submitted version.

## Funding

This research was funded by NIH grant AI062885, AI142570 and AI125434. TC was partially supported by the Jeane B. Kempner Postdoctoral Scholar Award from UTMB and Data Acquisition Grant from Institute for Human Infections and Immunity (IHII), UTMB.

## Acknowledgments

The authors would like to thank Drs. Leticia Castillo for providing samples of bronchial aspirates and Cynthia Tribble for assistance with submission of the manuscript.

## Conflict of interest

The authors declare that the research was conducted in the absence of any commercial or financial relationships that could be construed as a potential conflict of interest.

## Publisher’s note

All claims expressed in this article are solely those of the authors and do not necessarily represent those of their affiliated organizations, or those of the publisher, the editors and the reviewers. Any product that may be evaluated in this article, or claim that may be made by its manufacturer, is not guaranteed or endorsed by the publisher.
